# Supplementary material for: Intervallic intonation: Applying the Implication-Realization model of musical melody to speech intonation and prosody
Source: PLoS One. 2025 Nov 14;20(11):e0336780. doi: 10.1371/journal.pone.0336780 (PMC12617862; doi:10.1371/journal.pone.0336780)
Supplement: S1 Text — Analyzing “Happy birthday to you.”. (DOCX) [file pone.0336780.s001.docx]

## “Interval-based intonation: The Implication-Realization model of musical melody as representation of speech intonation and prosody”

### Alfred W. Cramer

# Supporting Information Text S1

## A musical demonstration of the Implication-Realization model (IR) Analyzing “Happy Birthday to You”

Fig A illustrates IR’s basic approach to implication, closure, and hierarchy by presenting a parsing of the song “Happy Birthday to You.” The analysis spans multiple levels, with all tones appearing on Level 1 and nearly all on Level 1a. The tones that do not initiate or close groupings are reduced on successive hierarchical levels until Level 4 retains only two tones. The initiating and closing notes of groupings are marked respectively by opening and closing bracket symbols ┌ and ┐. (All symbols are explained in the main article.) Above each note, arrows, tails, and commas trace the path toward closure, with arrows indicating increases in implication (expectancy) and tails visually "capturing" these arrows to represent decreases in expectancy.

**Fig A. IR parsing of Mildred J. and Patty Smith Hill, “Happy Birthday.”**

**
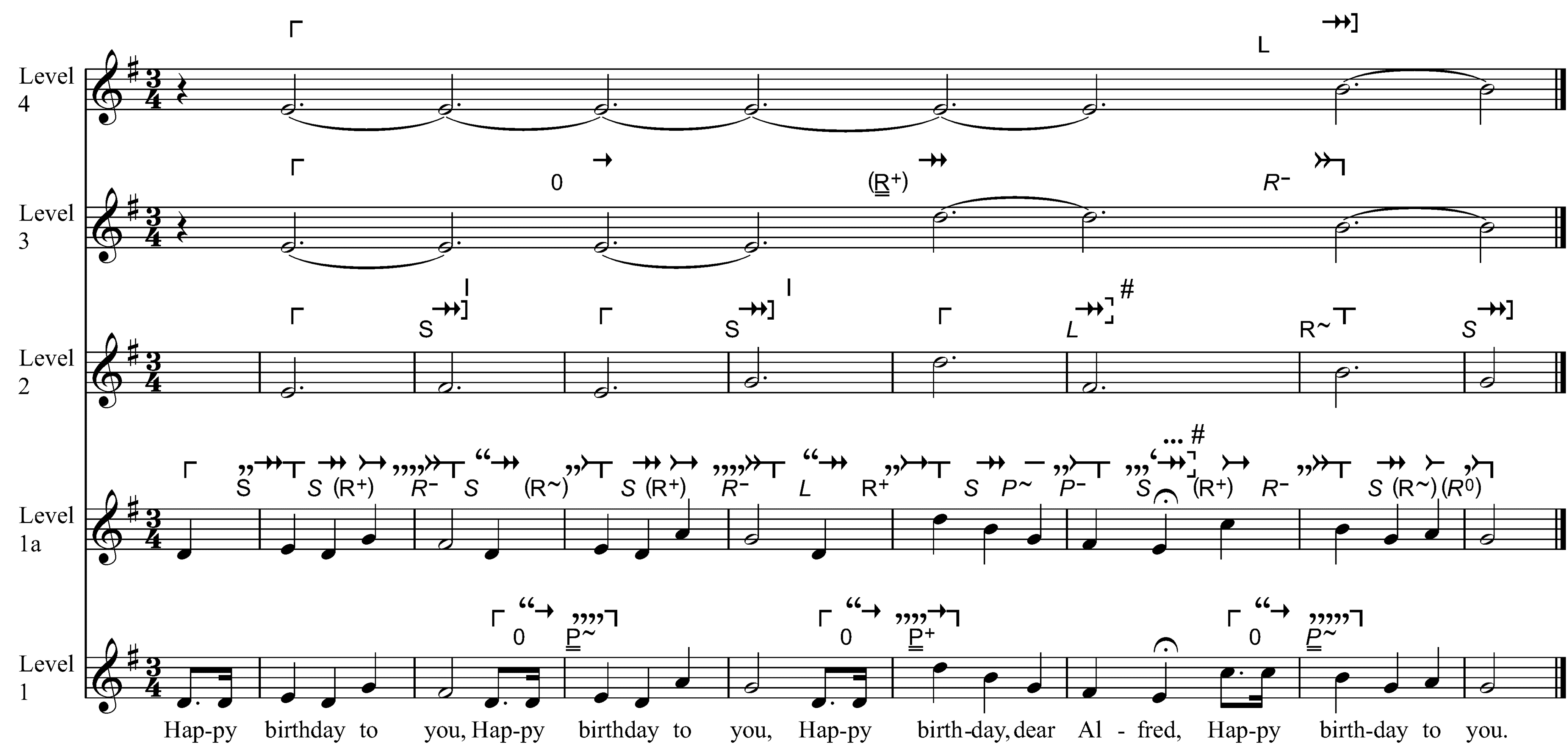
**

**Fig B. Detail of IR analysis of “Happy Birthday,” showing effects of intervals.** Arrows represent implication, or increased expectancy. Tails represent closure, or decreased expectancy. A comma shows the closural effect of downward motion.

**
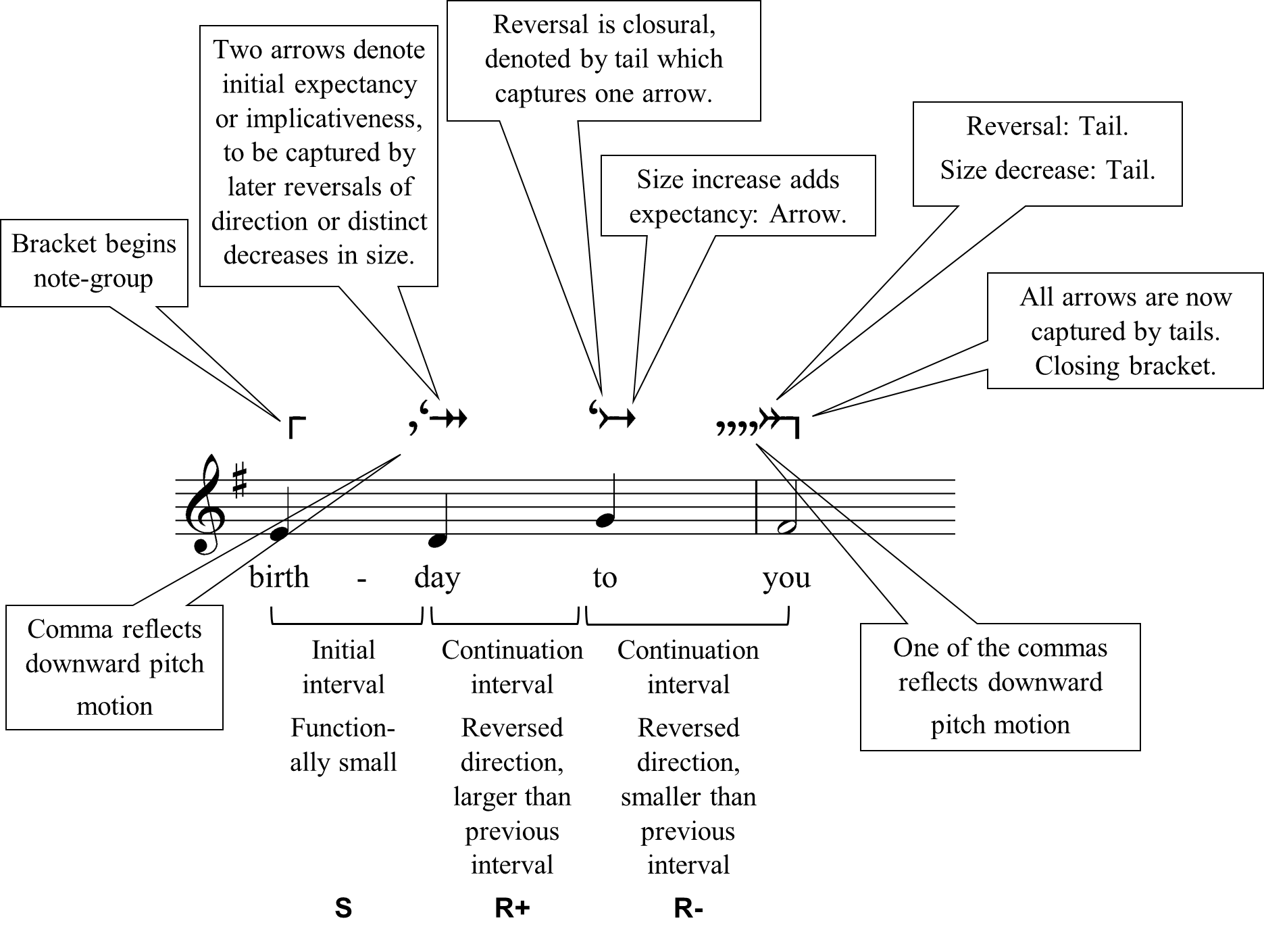
**

Fig B shows in a close-up how changes in implicativeness are correlated to IR’s representational scheme for pitch intervals. The first two tones (*birth*- and -*day*) together generate an implication, with the second tone carrying two arrows to represent implications of both direction and size. The next interval (from *‑day* to *to*) continues but reverses direction, reducing expectancy (as represented by a tail) while simultaneously gaining expectancy due to its larger size (as represented by an arrow). The final interval in this grouping (*to* to *you*) brings a double reduction in expectancy, represented by two tails, as it both reverses direction and presents a smaller interval. All arrows have now been captured, so the grouping is closed. Symbols succinctly identify the relevant interval comparisons: **S** for the small initial interval, **R+** for continuation with reversed direction and increased size, and **R-** for continuation with reversed direction and reduced size.

A number a commas are sprinkled into the figure as well. These denote *secondary* closural effects—that is, effects promoting closure that are not the result of comparative interval size or direction. Each comma adds a unit of closure equal to one tail to the tone to which it is attached. Such secondary effects will be discussed later, but here it may be observed that the comma on *-day* and one of the commas on *you* result from the downward pitch motion towards those tones, a closural effect independent of the chain of interval-size comparisons.

Most of the commas represent effects having to do with duration. While in Fig B they simply reinforce the effects of interval comparisons, Fig C shows how secondary effects can alter note-groupings. Secondary effects include not only the closural effects denoted by commas, but also nonclosural effects denoted by inverted commas [**‘**]. In this adaptation of IR, any closure contributed by a secondary feature is symbolized by a comma [**,**] equal to one tail, while any instability is symbolized by an inverted comma [**‘**] and is equal to one arrow. The tone on *‑py* is significantly shorter in duration than the preceding tone and is metrically weak, in that it does not fall on a beat. Each of these two factors is nonclosural, so it is marked with two inverted commas [‘]. The tone on which *birth-* falls, by contrast, has four times the duration of *‑py* (an increase so great that it receives three commas) and it also falls on the downbeat, a stronger metric position (giving it another comma). The four commas are weighed against the single arrow of cumulated expectancy and close the grouping. The earlier counterclosure represented by open-quotes on *‑py* is no longer in effect, since secondary features’ effects apply to only one tone.

**Fig C. Detail of IR analysis of “Happy Birthday,” showing secondary effects.** Commas and inverted commas show closure and counter-closure brought about by duration and meter.

**
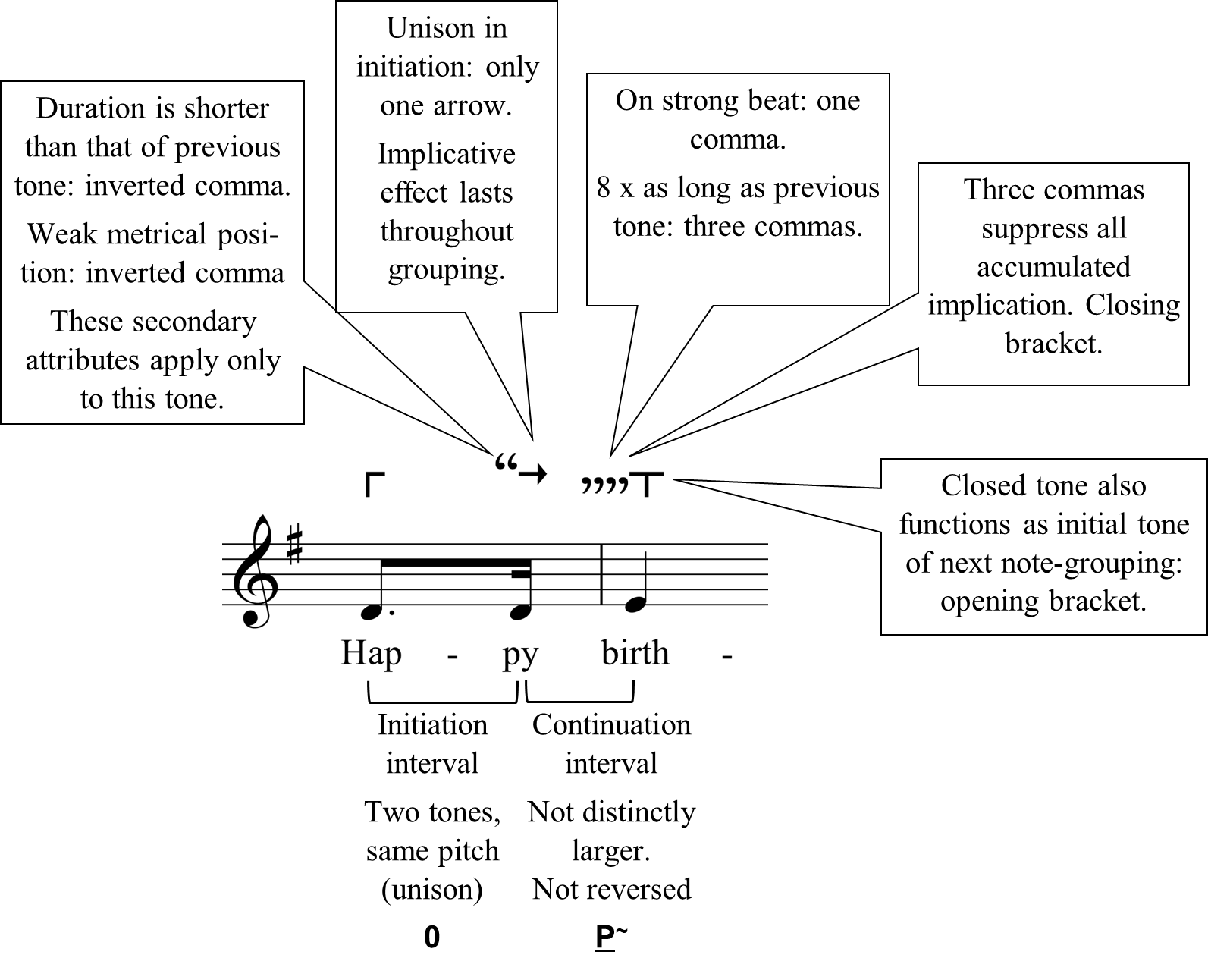
**

For comparison, Fig D shows the tune parsed without consideration of secondary features. The arrows and tails dictate closure on the bracketed notes, which then emerge in the Level 2 reduction. This parsing is clearly inadequate; one indicator of this is the conflict between the rhythm of Level 2 and the song’s clear meter.

**Fig D. IR analysis of “Happy Birthday” without secondary features.**

**
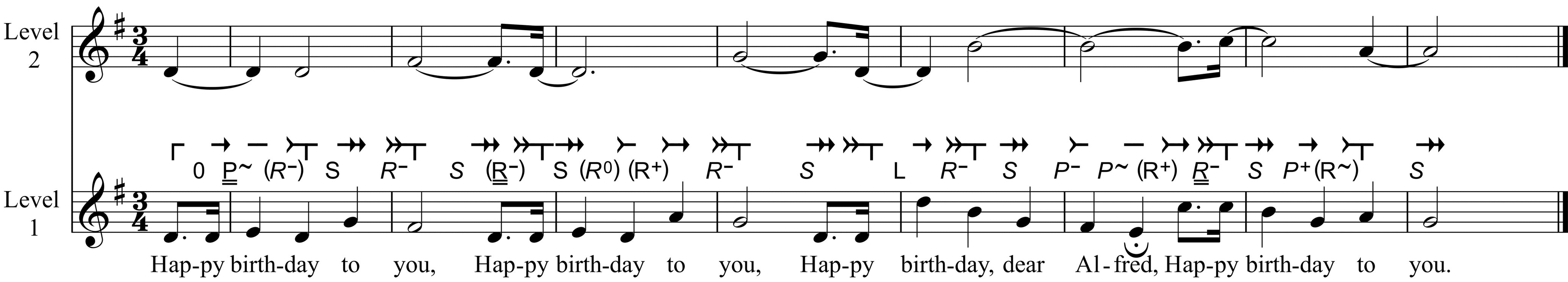
**

**Fig E. Details of IR analysis of “Happy Birthday.”** Suppression of implication brought about by the interpretation of a break.

**
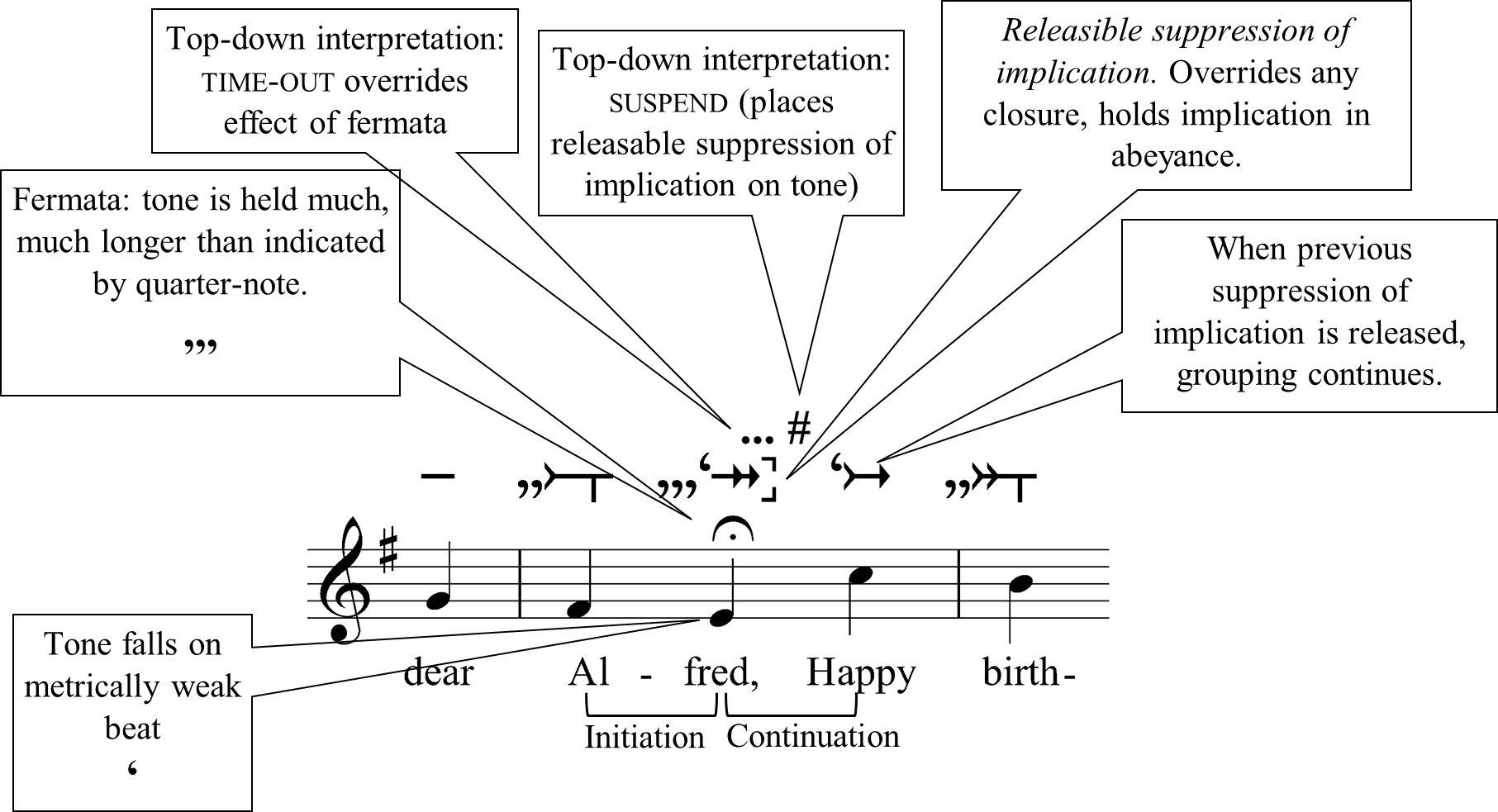
**

Fig E illustrates the concept of suppressed implication, a common occurrence in music. When singers hold the last syllable of *Dear Al-fred* for well over an extra beat, the listener understands that the melodic segment has been suspended, not closed. This is a top-down interpretation; in effect, the listener interprets the lengthening of duration on *‑fred* as a *suspend* [#], a type of break or pause that does not end the grouping but rather *releasably suppresses* the implication (as is denoted by a broken bracket). When the singers proceed to the next tone, the suppression is *released*, the implication becomes live again, and one hears *happy* as a continuation, not as a new beginning.

Breaks, then, provide articulations within phrases or larger statements by exploiting the power of implication to keep a grouping from coming to a full stop. Another type of break is seen in Level 2 of Fig A, where the first two statements of the words *happy birthday to you* are heard as though unfinished through the top-down imposition of a halt break [ | ]. This break permanently *suppresses* the accumulated implication (as symbolized by a square bracket), ends the grouping without closure, and casts the next tone as the beginning of a new grouping. (A somewhat different parsing of these levels might emerge through full attention to musical harmony that would be outside the scope of this study.)

Conventional approaches would emphasize the conclusiveness and stability brought about by the use of the song’s keynote pitch, G, for the final note of the tune. However, in this IR analysis, the final G closes groupings only on Level 1, and all G’s in Level 2 are implicative, resulting in the absence of this pitch from Levels 3 and 4. This disjuncture between melodic IR parsing and harmonically-framed analysis parallels the relationship between prosody and syntax in language. Much as the core tones of an intonational phrase generally highlight words other than syntactic heads, the core tones of a musical contour may differ from the tones whose pitches provide harmonic coherence.

IR analyses are often presented with the musical notation of the higher levels omitted, as in Fig F. The omitted notation can be fully inferred from the IR symbols. In parsing music, the omission saves space. In parsing speech, the omission is necessary due to the absence of a method for representing the pitch contours of higher hierarchical levels.

**Fig F. Condensed IR parsing of “Happy Birthday.”** Musical notation of higher hierarchical levels omitted.

**
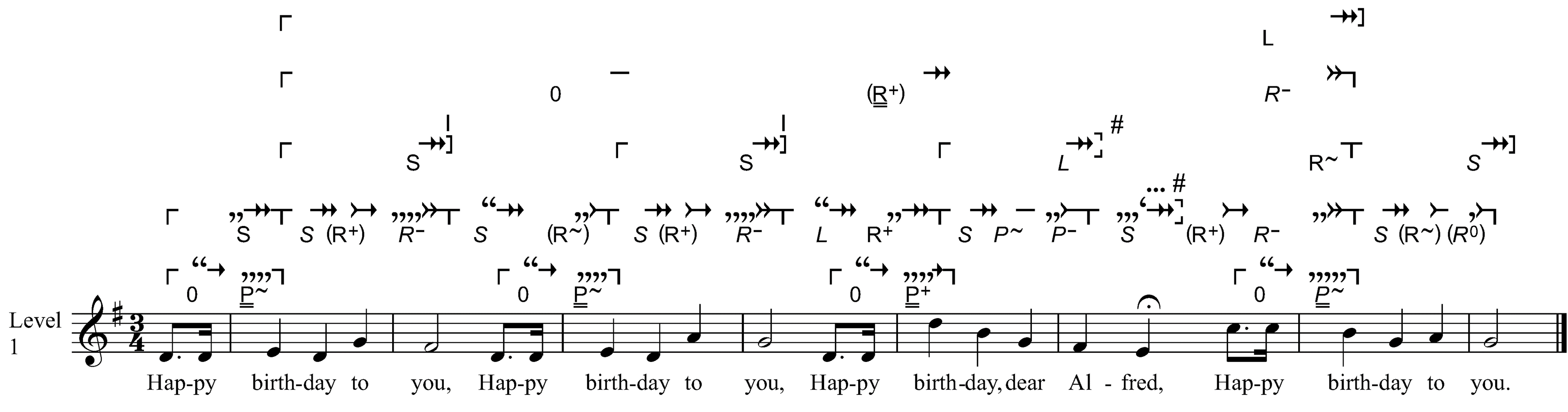
**

Readers familiar with IR will find the appearance of Fig F familiar. Such readers may, however, recognize a different set of labels for pitch intervals. Around 2000, Narmour revised the system he had used in earlier publications for labeling pitch structures based on interval comparisons [1–3]. Table A shows how the two labeling systems correspond.

**Table A. Correlation between different IR labeling systems**

| **Since 2000** | **Before 2000** |
| --- | --- |
| P^0^ or P^~^ | P |
| R- | R |
| D^0^ | D |
| P- | VP |
| P+ | IR |
| R^0^ | ID |
| R^~^ | IP |
| R+ | VR |

## References

1. Narmour E. The Analysis and Cognition of Basic Melodic Structures: The Implication-Realization Model. Chicago: University of Chicago Press; 1990.

2. Narmour E. The Analysis and Cognition of Melodic Complexity: The Implication-Realization Model. Chicago: University of Chicago Press; 1992.

3. Narmour E. Music Expectation by Cognitive Rule-Mapping. Music Perception. 2000;17: 329–399.
