# Supplementary material for: Intervallic intonation: Applying the Implication-Realization model of musical melody to speech intonation and prosody
Source: PLoS One. 2025 Nov 14;20(11):e0336780. doi: 10.1371/journal.pone.0336780 (PMC12617862; doi:10.1371/journal.pone.0336780)
Supplement: Attachment — Submitted filename: Response to reviewers.docx [file pone.0336780.s004.docx]

I thank the reviewers for their generous but candid comments. I’m especially grateful for the effort they put in to understand a long and involved article and the method associated with it.

Here I offer my responses to the points they raise:

*Both reviewers had concerns about the directness and specificity with which I stated the paper’s purpose and implications. Reviewer 1 said, “I thought it could be more direct and specific in identifying its implications. What sorts of specific problems in speech intonation identified in the literature could IR analysis potentially shed light on?” The other wrote, “One of the paper’s central claims is that IR‐generated parsings align with prosodic elements identified in the Autosegmental‐Metrical (AM) framework ‐‐ specifically, pitch accents, phrase accents, and boundary tones. While this alignment is intriguing, the paper does not explain why such agreement is meaningful or surprising. The AM framework is already a widely accepted and flexible system for representing intonation. If the IR model largely reproduces its structure, the question arises: what does the IR model add that AM does not already capture? Is the IR model merely a descriptive proxy, or does it offer theoretical or empirical advantages e.g., what exactly can it predict)?”*

I agree that the paper as originally submitted was vague in this respect. I have thoroughly rewritten the introduction as well as much of the rest of the paper to address these issues. Fundamentally, AM is good enough that a new model needs to align with its results, but there is room for a new model because AM leaves much unexplained in its parsings and in its theory. I have not found ways to make all of the following points clear in the paper, but I hope I stated enough of the following:

- AM and especially ToBI label tones quite selectively; IR can label them all and, within its hierarchical framework, posit the connections of each tone to others in the hierarchical network.
- It is acknowledged that paradigmatic and syntagmatic pitch coexist in intonation, but the latter is undertheorized in AM. IR proposes a fundamentally syntagmatic theory of the classication of pitch through interval size.
- Intonational features in AM are treated as aural prominences associated with a hierarchy of linguistic function (words to phrases; also the metrical prosodic grid). Approaches seeking to improve on AM—I name INTSINT and AM+ -- propose improvements in nomenclature and labeling rooted in the premises of AM, but they do not alter the fact that one must perform fairly extensive linguistic analysis before coming to the question of pitch. This is not necessarily wrong, but do we know? This paper shows that one can arrive at meaningful parsings by analyzing the spoken melodies themselves; the only linguistic pre-analysis needed is to segment the signals into syllables. Thus, not only does IR arrive at parsings rather similar to those of AM using a different theoretical basis, but it also does so using less linguistic information. (Or, if one views the treatment of breaks as essential, differently systematized information.)
- Besides its notion of prominence, AM does not offer an explanation of why the pitch accents, phrase accents, and boundary tones of intonation carry out their functions. These functions have particular appearances in IR parsings; that fact combined with IR’s model of implicative connection and hierarchy amounts to a theory that says boundary tones are implicative and unclosed (therefore setting up the next phrase) while pitch accents are deep-level tones with implicative connections to tones later in discourses (therefore marking the connections between key pieces of information). I have added statements about these issues in lines 155-161 (if “no markup” is selected in Track Changes), as well as early in the Discussion section and elsewhere.

*Further comments by Reviewer 1.*

*2) The Tables need to be revised:*

*a) they are incomplete. They don't contain all of the various symbols contained in the analyses (e.g., tilde [~] and number sign [#]). They must be carefully collated with the symbols in the figures and the body text.*

*b) the symbols in the tables are not consistent with those in the analysis. For example, the curly brackets shown in Table 1 don't appear in the analyses.*

*c) I assume the question marks (?) throughout Table 2 are a forma􀆫ng error with the file, and not actual symbols. (See also, "Error! Reference source not found" in Table 2.) All of these issues make it a chore to read through the analyses. For example, I really had to dig to know what R~ means on l. 559. I strongly recommend that the author consider the reader here. A "one‐stop shopping" table where the reader can look up any unfamiliar symbol in the analyses would be helpful.*

I apologize. All of these issues come down to formatting errors, mostly related to the fact that some of the symbols (such as arrowtails) don’t exist in any standard font so I created one myself. I hope to have addressed all of these errors. I have removed virtually all unusual symbols from the running text, and in consultation with members of PLOS One’s editorial staff I have changed what the reviewer calls “one-stop” tables (actually, a pair of tables) into graphic figures so that all of the symbols display correctly.

*3) Although I did eventually read the whole thing, the author might benefit from knowing that, at some point on my first pass, I stopped reading the (very long) introduction and skipped to the data section. I anticipate many busy readers might do the same. For this reason, the author might consider moving some Introduction material to supplementary materials (e.g., ll. 193‐274) and pointing the reader to those materials for further reading. Other solutions are possible (e.g., just trimming the Intro text).*

I have moved the original’s ll. 193-274 (which show how to do an IR analysis of “Happy Birthday”) to Supporting Information. At the editor’s request, I have replaced it with a walk-through showing illustrating the creation of an IR parsing, using “Marianna made the marmalade” as the example. This is more clearly part of Method rather than Introduction now.

While I have shortened some of the introduction, I have also added some things to address concerns. That means the introduction is not shorter, but I hope that by being more on point it doesn’t seem as tedious. I have also improved the section headings throughout the article to guide readers who feel the need to move ahead.

*Some other minor concerns:*

*1) l. 129‐131 ‐ Please provide supporting evidence for this empirical statement. Some contradicting evidence:*

*‐ Ammirante & Russo (2023), who showed that, whereas listener expectancy ratings on instrumental melodies were better predicted by relative pitch, ratings on sung melodies were better predicted by absolute pitch.*

*‐ Jacoby et al. (2019) showed that singers’ reproductions of sequences of tones tend to be distributed around their absolute pitch heights rather than transpositions that preserve their pitch relations*

I made this statement about transposition of musical melody as part of the argument that syntagmatic pitch matters. Since it is about music, it’s not very good support for this claim, so I have removed it. Thank you for the citations. I have actually added a citation of Ammirante and Russo to the article (in the general discussion of IR). I’m not sure Jacoby et al. say what you claim here; in fact, they conclude that interval size (or “logarithmic scales for pitch”, as they say) are *preserved* even when a culture doesn’t have octave equivalence. In any case, I don’t want to get too deep into the question of absolute (or merely paradigmatic) pitch vs. syntagmatic, since I don’t think it’s controversial to claim that the two coexist. IR is fundamentally syntagmatic (with paradigmatic available only as a top-down factor), and if it proves successful in analyzing speech that is a useful data point for the future.

*2) l. 184. Did Margulis really "introduce" the term "expectancy"? Not sure if Meyer used "expectancy" or "expectation". A search of google scholar shows the term "melodic expectancy" goes back at least as far back as Carlsen 1981. Or maybe I'm misinterpreting what you mean by "introduced"?*

Thank you for catching that. This was like learning a new word and suddenly discovering that everyone else has been using it all the time. Yes, of course Margulis didn’t introduce the term (although she contributed importantly to the understanding of its musical significance). It’s been used prominently since the very earliest empirical studies of Narmour’s model. I’ve removed the statement.

*3) ll. 323‐327. Not sure I follow the logic here (l. 325: "making it likely that [in speech] there are meaningful distinctions between intervals of very small sizes"). Aren't listeners constrained by JND for pitch? Aren't speakers constrained by intonation accuracy? E.g., studies from Pfordresher and Brown of singing without a pitch referent ‐ a good analogue for pitch in speech ‐ show that inaccurate and imprecise production of intervals are the norm. Also, if very small intervals are meaningfully distinct in speech, wouldn't we expect tone languages to have more tones than musical scales?*

*4) Related to 3), I found myself wanting to know the size of at least some of the speech intervals described in the analyses. The reason is that I'd like to be able to critically evaluate for myself claims of meaningfulness attributed to small speech intervals, which alternatively might just be meaningless variability in speech F0 production. This could be accomplished by having cents (or semitones) instead of Hz on the y‐axis in the Praat screenshot, although I recognize that Praat might not do this natively.*

I have clarified the problematic lines 323-327; see ll 421-430 in the revision. I have also added pitch values in semitones (with reference to 100Hz) to all of the analyses. To the larger issue: IR’s categorization of intervals accounts for inaccuracy and imprecision in that the most significant interval categories (those leading to labels of + or -) involve interval-size differences greater than something like 3 semitones. I have kept the zero-size initial interval and the zero-size interval comparison in order to remain consistent with Narmour’s model. The difference between R0 and R~ or P0 and P~ makes no difference in parsing, while the difference between an initial 0 and an initial S is that 0 receives only one arrow instead of two. Perhaps the thresholds should be set higher than 0.2 semitones, but they should be set separately, and for that I would have to do some recoding of IRProsodyParser. I’m afraid that will have to wait.

*5) for readers interested in potentially using IRProsodyParser, its description on pp. 431‐436 could be more clear in terms of what it does and doesn't do. The author might consider describing it as semiautomated, and specify what needs to be done manually. I had to download and test it out to know.*

Thank you for testing the scripts. As noted above, I have added that clarification as well as a narrative of the creation of an analysis. See the sections *Method: Parsing Prosody Using IR* and *Analyzing with IRProsodyParser: a demonstration*. (They begin at line 219.)

*6) ll. 485‐488. Why not define the target pitch consistently? Also, please clarify whether it is the algorithm (automated) that is picking and choosing between average, local minima, etc., or the user manually making this decision.*

The user is manually making the decision. I have clarified and amplified the explanation in lines 274-293.

*Typos, etc.:*

I have corrected the typos and other mistakes. Here I will comment on the ones that involve matters of substance.

*‐ ll. 526‐527: maybe so for the discrete pitch (long grey bars) but not for the raw contour (black line).*

*For the raw contour, the pitch of "the" looks to be higher than for "mar".*

Since I think many readers may have this question, I have amplified the discussion in and around ll. 284-289.

*‐ l. 743: I don't know what is meant by "mimetic" in this context*

I’ve changed the phrase that follows so that it reads as a definition. I considered removing the word “mimetic” but I’ve kept it because it appears in some theoretical discussions of intonation and information.

*Further comments by Reviewer #2*

*2. Limits of Musical Analogies in Speech Prosody: A more fundamental concern relates to the underlying assumption that principles derived from music perception (e.g., implication, realization, and transposability) can be directly ported to speech prosody. While both music and speech involve pitch, their functional and perceptual constraints differ. Musical melodies often assume transposability across registers or instruments, but speech prosody is anchored to a speaker’s modal pitch, with pitch variation serving different pragmatic and grammatical functions. The model does not appear to address how these vocal constraints may affect the viability of IR principles as applied to prosody.*

Reviewer #1 had a related question related to the issue of whether speech pitch is absolute or transposable; see my response to that question, above. I lapsed by bringing up the issue of transposition at one point in my original submission. That may have sent a false implication about what I am trying to say. I believe it is sufficient to note that paradigmatic and syntagmatic aspects of pitch exist in speech, and that IR contributes to a needed theorization of the syntagmatic. There are larger issues here, of course. It is true that “speech prosody is anchored to a speaker’s modal pitch”—but what that means, I believe, is that within the speaker’s comfortable pitch range, pitch targets (paradigmatic) and pitch intervals (syntagmatic) are both relevant. It has been noted in the literature (von Hippel & Huron; Ammirante & Russo) that musical singing is constrained by vocal range, but that means that interval matters less than pitch height *when near the extremes of the vocal range*. Certainly there will be limits to what IR can show, but we need to try the model in order to find out what they are.

*3. Lack of Empirical Validation: Although the paper introduces the IR model as “cognitively based,” it does not engage with existing empirical literature supporting the psychological reality of hierarchical structures as described by IR. For example, in musical perception, Narmour’s IR model has received both support and critique in experimental studies. However, no such empirical link is drawn here for speech. The parsing examples (e.g., Figure 2) are intuitive, but without listener data or perceptual testing, it’s unclear whether the proposed hierarchical structures correspond to actual prosodic grouping by human listeners.*

I agree that empirical support is ultimately necessary, and I intend this paper to build toward the possibility of experimental studies. Actually carrying out the empirical research is beyond the already large scope of this paper. Experimental work on IR has focused almost entirely on claims about expectations following large versus small intervals, leaving other crucial aspects of the model unexplored. I am convinced that a major reason for this is that it is unclear what an IR analysis says or shows. I offer the analytically-framed adaptation of the theory partly in hope that experimenters will find ways to investigate claims about closure, categorization, and the like. As a music theorist, I find the application to speech more obvious than the application to music; I also find that it is helping me to understand what IR accomplishes for music.

The adaptation of the symbology in this paper is, in the long run, intended to make it clearer what IR shows. So is the application to language. IR’s basic assumption about bottom-up pitch processing is that it is unencumbered by musical harmony. In that sense, one may expect it to be better suited to spoken pitch than to music, provided that its assumption about alignment of pitch targets to syllables holds up. For now, I believe that assumption is believed by many linguists to be reasonable. In the long run, IR might provide a framework to test it.

By offering a sense of what IR may show about speech, I hope to prompt experimental studies related to both speech and music. I would agree that the needs you articulate in this comment are real, but that if we attempt to build models like IR by empirically testing all of the basic building blocks we will never get to the model, and then we will not know which building blocks need to be tested. The work needs to be done in both directions. The existing experimental work on IR was done *after* Narmour had presented an extensive, analysis-based theoretical framework for IR in music.

Back to the manuscript. In the revision, I have not lengthened the discussion of the existing empirical literature about IR, but I have referred to a few more studies, and said more about them. I have added a discussion of ‘t Hart 1981 (lines 424ff), intonation-based empirical work that directly supports IR’s interval-categorization scheme. See lines 1956-213. I have attempted to improve the discussion of empirical validation of this application of IR to speech in the first few paragraphs of the Discussion section.

*4. Scope and Suitability for Journal Venue: While the paper may be a valuable contribution to methodological work in prosody, the format feels more like a theoretical exposition or tool introduction than an empirical study. For a journal like PLOS ONE, which emphasizes scientifically valid contributions, it would be helpful to include at least a minimal test of the IR model’s value. For example, a comparison with AM‐based annotations on listener perception of prosodic boundaries, or a computational evaluation of model accuracy. In its current form, the work may be better suited to a journal focused on phonetics or theoretical linguistics.*

For various reasons, I concluded that PLOS One was a good venue for this work, but I leave that decision up to the editor. I agree that my original submission read more like “a theoretical exposition or tool introduction.” I hope that this revision makes the article’s purposes clearer.
