# Supplementary material for: Intervallic intonation: Applying the Implication-Realization model of musical melody to speech intonation and prosody
Source: PLoS One. 2025 Nov 14;20(11):e0336780. doi: 10.1371/journal.pone.0336780 (PMC12617862; doi:10.1371/journal.pone.0336780)
Supplement: Attachment — Submitted filename: Response to reviewers oct2025.docx [file pone.0336780.s005.docx]

Author’s response to reviewer comments
PONE-D-25-17112R1
Intervallic intonation: applying the Implication-Realization model of musical melody to speech intonation and prosody

The following comments were relayed to me:

I think there is still room to make the paper more amenable to a non-linear reader that wants to first quickly scan the paper for information related to their research interests before committing to reading from beginning to end: -

- one or two "roadmap" sentences at the end of the last paragraph of the Introduction that specify what the method and case studies will accomplish
- where possible, the findings reviewed in the Discussion should be explicitly linked to a case study and/or figure (e.g., "(see Figure X)". For example, it looks like this could be done for the sentence beginning on line 766.

I agree with this. As the cover letter also notes, my new revision includes the following changes:

- Added a few sentences giving the roadmap of the article, and grouped them with the previous final sentence of the article’s first paragraph. Together, all of these sentences now constitute the second paragraph of the article, which begins on line 49.
- Added about a dozen in-text references to figures and adjusted the text surrounding a few more, as a way to help the reader follow.
